# Quantitative assessment and optimization of parallel contact model for flexible paddy straw: a definitive screening and central composite design approach using discrete element method

**DOI:** 10.1038/s41598-024-52388-7

**Published:** 2024-01-23

**Authors:** Abhishek Patel, Krishna Pratap Singh, Ajay Kumar Roul, Rohit Dilip Nalawade, Aman Mahore, Mohit Kumar, Prasad Avilala, Chelpuri Ramulu, Berhanu Kebede, Abhik Patra

**Affiliations:** 1 ICAR- Central Institute of Agricultural Engineering, Bhopal, India; 2 ICAR Krishi Anusandhan Bhawan II, New Delhi, India; 3 Agricultural Mechanization Division, ICAR- Central Institute of Agricultural Engineering, Berasia Road, Nabibagh, Bhopal, India; 4 Sri Karan Narendra Agriculture University, Jobner, Jaipur, India; 5 Altair Engineering, Bangalore, India; 6 KVK Madhopur, Rajendra Prasad Central Agricultural University, Pusa, Samastipur, Bihar India; 7 Department of Agricultural Engineering, School of Agricultural and Food Engineering, Ambo University, Ambo, Ethiopia; 8 KVK Narkatiyaganj, Rajendra Prasad Central Agricultural University, Pusa, Samastipur, Bihar India

**Keywords:** Computational science, Engineering, Materials science

## Abstract

To simulate the bending behaviour of paddy straw at varied moisture contents after crop harvesting, we created a flexible paddy straw specimen model based on the Hertz–Mindlin with parallel contact bonding model using the discrete element model (DEM) approach. The research presented in this study aims to investigate a new approach called Definitive Screening Design (DSD) for parameterizing and screening the most significant parameters of the DEM model. This investigation will specifically focus on the three-point bending test as a means of parameterization, and the shear plate test will be used for validation purposes. In addition, the most influential DEM parameters were optimized using another Design of Experiments approach called Central Composite Design. The findings from the DSD indicated that parameters such as bonded disk scale, normal stiffness, and shear stiffness have the highest impact on the bending force, while the coefficient of static friction (Straw-Steel) has the least effect. The three bonding parameters were respectively calibrated with the loading rate (0.42, 0.5, and 0.58 mm s^−1^) and a good agreement between actual and simulated shear force at moisture content M_1_—35 ± 3.4%, M_2_—24 ± 2.2% and M_3_—17 ± 2.6%. Modelled stem helps simulate the straw with low error and increases the accuracy of the simulation. The validated model, with an average relative error of 5.43, 7.63, and 8.86 per cent, produced reasonable agreement between measured and simulated shear force value and loading rate.

## Introduction

Paddy (*Oryza sativa L*.) is one of the cereal grains that provides food for more than 50% of the world’s population^1^. Paddy straw management is a major problem in combined harvested paddy fields during the sowing process of paddy-wheat rotation^2^. It has been suggested that in northwest India, the inclusion of straw 15–20 days before wheat sowing should replace paddy straw burning and resolve the problem by straw chopper cum incorporator^3^. Testing the straw incorporation machine and its modification in a short time is a challenging process, and the realistic development of the DEM straw model can solve this problem. Numerical simulations can provide quick forecasts, eliminating the need for significant field testing^4^. Discrete Element Modelling (DEM) is a numerical method for simulating the motion of a group of particles that collide with one another^5^. It is revealed that the development of a straw model has been a novel approach in DEM research because of the complexity of straws' physical structure^6^. Some related studies already developed a straw model by different methods and used the simulation to predict many macroscopic behaviours. To calibrate variables including Young’s modulus, cohesion damping coefficient, and elasticity modulus, they performed cantilever beam and three-point bending experiments^7–9^. The processes of cutting rattan straw and maize straw scarfskin, respectively, to get insights into straw-cutting behaviour^10,11^. Creating a standard ramie stalk bending model through material experiments and finite element method simulations provides a vital foundation for designing efficient harvesting equipment^12^. Analytical model (AM) with the Discrete Element Method (DEM), highlighting DEM’s superior performance in predicting cutting forces and soil profile changes^13^.

Nowadays, the applications of DEM in agriculture are much more popular, but the research with flexible crop stems at different moisture content in DEM is scanty. The researcher considered the bendable crop stems from taking the segment shapes of spheres, cylinders, or capsules, and all these have linear elastic bending behaviour^14,15^. To figure out the accurate result of simulation with calibration parameters of factor sensitive analysis and different statistical designs utilizing physical and different virtual tests such as three-point bending test, angle of repose test by split pipe method, compression test, and pendulum type bending test for real-time deformation rates were revealed^15–17^. In the study, the mechanical behaviour of elastic fibers and innovative bonded-sphere and sphero-cylinder DEM models has been planned in current times, and the result was analyzed to perform a study for a dynamic threshing process by hollow cylindrical elastic bond based on optimization of threshing unit^8,18^. With the assistance of a parallel bonding model in DEM and the calibration of bonding parameters under Hertz–Mindlin^9^, proposed a study to simulate the bending behaviour of flexible wheat straw and developed a switch grass model using the uniaxial compression model^19^. A systematic methodology was employed, as outlined by^7^ in the bending test and cantilever test, followed by the uniaxial compression test as per^20^.

The force measurements obtained from the bending test can be used to evaluate the quality and durability of the paddy straw, as well as to assess its performance in various applications. This information is important for researchers, engineers, and agricultural practitioners involved in designing and developing agricultural machinery, processing techniques, and storage methods that handle paddy straw effectively and minimize any potential damage or loss during handling and transportation. This will help us to develop a well-behaved model of single straw specimens in DEM and further use it to analyze the data required for the incorporation of DEM. So far, there is no such model for straw calibration at different moisture levels concerning different loading rates, and limited literature is available.

The goals of this study are as follows: 1. To perform a screening experiment aimed at identifying and characterizing the key DEM parameters that impact the bending force in three-point bending simulations. 2. To refine the crucial DEM parameters through physical three-point bending tests and CCD experiments at three different moisture content. 3. To validate the newly developed DEM model by conducting a shear plate test.

## Material and methods

### Discrete element modelling (DEM)

Recent advancements in computer technology have facilitated the utilization of DEM for simulating granular materials in industrial and laboratory settings^21^. This methodology entails several sequential steps condensed into a single time step, typically involving the following four stages:

(1) Initial preparation, which includes calculating particle field attributes, size, and arrangement; (2) Defining the physical characteristics of particles (e.g., mass, motion behaviour, particle interactions, and failure criteria) and applying boundary conditions; (3) Computations for determining particle forces, velocities, accelerations, and positions; (4) Post-processing to generate simulated particle images. These stages collectively constitute the DEM process.

### Modeling of the flexible paddy straw

The paddy straw was developed by adding the multi-spheres in meta particles. With a parallel contact model, Hertz–Mindlin was used to develop flexible paddy straws. Bonding properties were selected such that the developed model can represent the paddy straw’s actual behaviour. The sphere’s diameter defines the straw diameter selected according to the average diameter of paddy straw in the physical experiment.

#### Flexible straw model

A solid sphere (diameter: 5 mm, straw length: 80 mm) was obtained by considering the shape of the paddy straw, accounting for the overlap between two spheres and having a given contact radius. Uniformly filled the straw unit with 60 spheres using Altair EDEM 2021 software (DEM Solutions Ltd., Edinburgh, UK).

**Figure 1:**
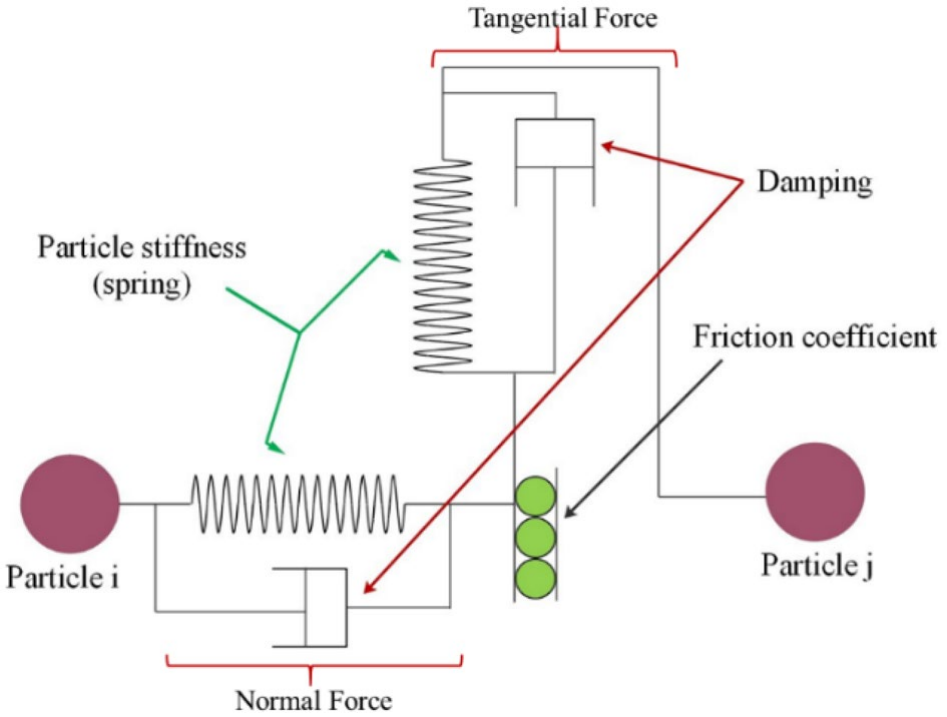
Schematic diagram of Hertz Mindlin contact model.

The Hertz–Mindlin (no-slip) contact model was used in the straw model as default in the EDEM programme. Due to its inherent benefits in terms of accuracy and effectiveness, as shown in Fig. 1, this model is preferred. The tangential force component of this particular model was based on research^22^, whereas the normal force component was calculated using the Hertzian contact theory. It is noteworthy that damping elements are present in the normal and tangential forces, with the related recovery and damping coefficients. The total normal force is made up of both the normal force component and the damping normal force component in the context of the Hertz–Mindlin (no-slip) contact model.

1 $${F}_{n}={-K}_{n}{\updelta }_{n}+ {C}_{n}{v}_{n}^{\underset{rel}{\to }}$$

where K_n_, C_n_ and ${v}_{n}^{rel}$ are the spring constant, damping coefficient and relative velocity in the normal direction, respectively. To model flexible paddy straw within the DEM framework, several sphere units were organized and interconnected using the Hertz–Mindlin with parallel contact V2 bonding model and these forces and moments can be computed using Eqs. (2)–(4)^23^.

2 $$\Delta F_{n}^{B} = - v_{n}^{{\to _{rel} }} \cdot k_{nb} \cdot A \cdot \Delta t$$

3 $$\Delta F_{t}^{B} = - v_{t}^{{\to _{rel} }} \cdot k_{tb} \cdot A \cdot \Delta t$$

4 $$\Delta M_{n}^{B} = - \omega_{n}^{{\to _{rel} }} \cdot k_{nb} \cdot A \cdot \Delta t$$

5 $$\Delta M_{t}^{B} = - \omega_{t}^{{\to _{rel} }} \cdot k_{tb} \cdot A \cdot \Delta t$$

6 $$\sigma_{critical} < \sigma_{max} = \frac{{ - F_{n}^{B} }}{A} + \frac{{2M_{t}^{B} }}{J}R_{B}$$

7 $$\tau_{critical} < \tau_{max} = \frac{{ - F_{t}^{B} }}{A} + \frac{{2M_{n}^{B} }}{J}R_{B}$$

where ${v}_{n}^{rel},{v}_{t}^{rel}$ and ${\omega }_{n}^{rel},{\omega }_{t}^{rel}$ are the relative and angular velocities ($n$ and $t$ represent the normal and tangential directions); ${\Delta }_{t}$ time step; ${k}_{nb}$ and ${k}_{tb}$ are the normal and shear stiffness per unit area mentioned in Eqs. (2)–(5).

In addition, the flexible straw would be broken when the maximum normal or tangential bonding stresses (σ_max_, τ_max_) exceeded their critical values (σ_critical_, τ_critical_).

### Definitive screening design (DSD)

As suggested by Favre and Chaves in 2021, the DSD is a revolutionary statistical screening technique that makes it possible to examine the impact of four or more process factors on one or more response variables while needing fewer runs than conventional screening designs. Consequently, the input factors employed in DEM software simulations underwent a parametric evaluation to explore their impact on responses and identify the most influential parameters for subsequent calibration. This study utilized a Design of Screening Experiments (DSD) approach to conduct parametric assessments on twelve distinct input parameters. These parameters encompassed various characteristics such as the shear modulus of straw, restitution coefficient between straw and straw, static friction coefficient between straw and straw, rolling friction coefficient between straw and straw, restitution coefficient between straw and steel, static friction coefficient between straw and steel, rolling friction coefficient between straw and steel, normal stiffness, shear stiffness, normal strength, shear strength, and bonded disk scale.

Table 1 outlines the operational ranges of these parameters, which are selected based on a comprehensive literature review and prior research experiences. The straw’s Poisson’s ratio was predetermined at 0.3, as suggested by^24^, and was therefore considered a constant, not subject to variation in the DSD. However, the minimum run size for DSD rose by four runs as a result of the addition of two extra variables, leading to an expanded DSD with an actual total of 29 runs (Table 2)^25^.

**Table 1:** Selected input parameters and their ranges for DSD.

Factors	Units	Symbols	Levels		
			Low (− 1)	Middle (0)	High (+ 1)
Coefficient of restitution (straw-straw)	–	CR (straw straw)	0.2	0.4	0.6
Coefficient of friction (straw-straw)	–	CF (straw straw)	0.2	0.55	0.9
Coefficient of restitution (straw-steel)	–	CR (straw steel)	0.2	0.4	0.6
Coefficient of friction (straw-steel)	–	CF (straw steel)	0.3	0.55	0.8
Coefficient of rolling friction (straw-steel)	–	CRF (straw steel)	0.01	0.035	0.06
Normal stiffness	Nm^−3^	–	1 × 10^8^	5.5 × 10^8^	1 × 10^9^
Shear stiffness	Nm^−3^	–	1 × 10^8^	5.5 × 10^8^	1 × 10^9^
Normal strength	Pa	–	1 × 10^6^	5.5 × 10^8^	1 × 10^9^
Shear strength	Pa	–	1 × 10^6^	5.5 × 10^8^	1 × 10^9^
Bonded disk scale	–	BDS	3	4	5

**Table 2:** Experimental design matrix for DSD.

Runs	SM (straw)	CR (straw-straw)	CSF (straw-straw)	CRF (straw-straw)	CR (straw-steel)	CSF (straw-steel)	CRF (straw-steel)	Normal stiffness	Shear stiffness	Normal strength	Shear strength	BDS
1	1E+07	0.6	0.9	0.05	0.6	0.3	0.01	1E+09	1E+08	5.01E+08	1E+06	5
2	1E+06	0.2	0.9	0.03	0.6	0.3	0.06	1E+09	1E+08	1E+06	1E+06	3
3	1E+06	0.6	0.2	0.01	0.2	0.3	0.06	1E+09	1E+08	1E+09	5.01E+08	5
4	1E+06	0.2	0.2	0.01	0.6	0.8	0.01	1E+09	5.5E+08	1E+09	1E+06	5
5	1E+06	0.6	0.2	0.05	0.6	0.3	0.01	1E+08	1E+08	1E+09	1E+09	3
6	5.5E+06	0.2	0.2	0.01	0.2	0.3	0.01	1E+08	1E+08	1E+06	1E+06	3
7	1E+07	0.2	0.2	0.05	0.2	0.55	0.01	1E+09	1E+08	1E+06	1E+09	5
8	1E+06	0.6	0.9	0.01	0.2	0.3	0.01	1E+09	1E+09	1E+06	1E+09	4
9	1E+07	0.2	0.9	0.05	0.6	0.8	0.01	1E+08	1E+09	1E+06	5.01E+08	3
10	5.5E+06	0.6	0.9	0.05	0.6	0.8	0.06	1E+09	1E+09	1E+09	1E+09	5
11	1E+06	0.2	0.2	0.01	0.2	0.8	0.06	1E+08	1E+09	5.01E+08	1E+09	3
12	1E+07	0.6	0.2	0.01	0.6	0.3	0.035	1E+08	1E+09	1E+06	1E+06	5
13	1E+07	0.6	0.2	0.03	0.2	0.8	0.01	1E+08	1E+09	1E+09	1E+09	5
14	1E+06	0.4	0.9	0.01	0.6	0.8	0.01	1E+08	1E+08	1E+06	1E+09	5
15	1E+06	0.2	0.2	0.05	0.6	0.3	0.06	5.5E+08	1E+09	1E+06	1E+09	5
16	1E+07	0.6	0.9	0.01	0.2	0.8	0.01	5.5E+08	1E+08	1E+09	1E+06	3
17	1E+06	0.6	0.9	0.01	0.6	0.55	0.06	1E+08	1E+09	1E+09	1E+06	3
18	1E+06	0.2	0.9	0.05	0.2	0.8	0.035	1E+09	1E+08	1E+09	1E+09	3
19	1E+06	0.2	0.9	0.05	0.2	0.3	0.01	1E+08	1E+09	1E+09	1E+06	5
20	1E+07	0.6	0.9	0.05	0.2	0.3	0.06	1E+08	5.5E+08	1E+06	1E+09	3
21	1E+06	0.6	0.2	0.05	0.4	0.8	0.01	1E+09	1E+09	1E+06	1E+06	3
22	1E+07	0.2	0.9	0.01	0.4	0.3	0.06	1E+08	1E+08	1E+09	1E+09	5
23	1E+06	0.6	0.55	0.05	0.2	0.8	0.06	1E+08	1E+08	1E+06	1E+06	5
24	1E+07	0.4	0.2	0.05	0.2	0.3	0.06	1E+09	1E+09	1E+09	1E+06	3
25	5.5E+06	0.4	0.55	0.03	0.4	0.55	0.035	5.5E+08	5.5E+08	5.01E+08	5.01E+08	4

### DEM model of three-point bending test

**Figure 2:**
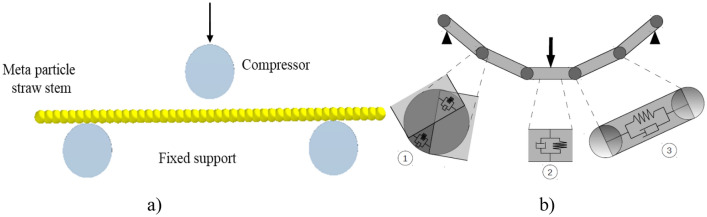
A bendable stem of crop (**a**) Placement of meta particles-based straw on three-point support (**b**) Representation of spring and mass damper between two particles^15^.

To better understand the flexibility of straw, the three-point bending test DEM model was simulated using the Altair EDEM 2021 software, and a setup for the test was included in the straw model (Fig. 2a). Using Kelvin-Voigt models, these two spheres were connected by various linear, parallel spring-damper systems (Fig. 2b). The bending stiffness of the joints is examined through the stiffness (kb) and placement of the springs. At the joints, dampers (cb) prevent oscillations and guarantee simulation stability, while the straw is subjected to a normal force at the support point. To account for this, an extra set of Kelvin–Voigt systems (kr and cr), specifically in the radial direction (component 2 in Fig. 2b), is included per segment and joint.

An additional spring (kt) and damper (ct), both of which are coupled to the two discs within a specific segment (Fig. 2a), are responsible for the tensile stiffness of each segment. As shown in Fig. 2b, the parallel contact version 2 and hysteretic spring contact model are used in the 3-point bending simulation. The flexibility and plasticity properties of the material are considered in this model.

**Figure 3:**
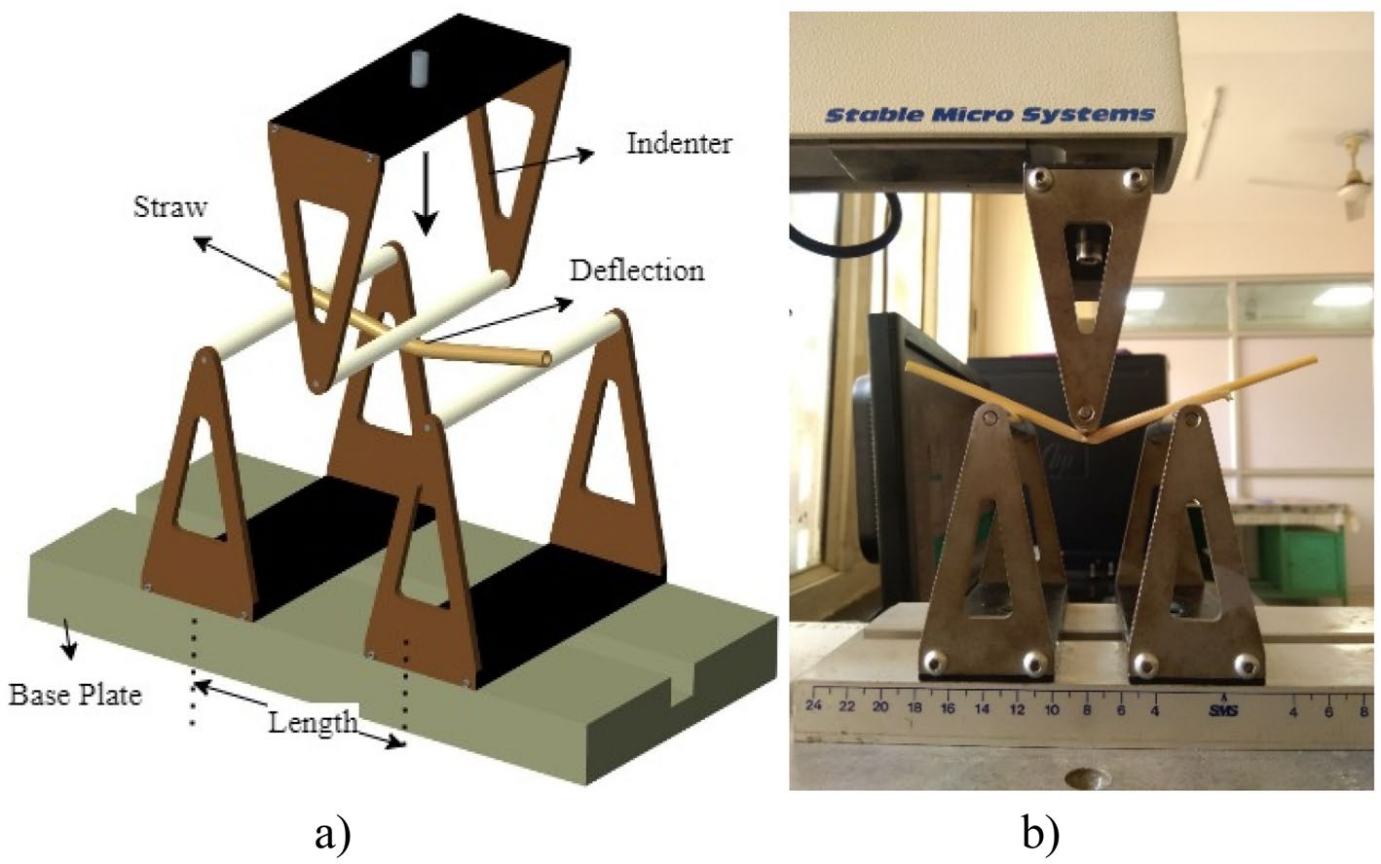
Three-point bending test (**a**) Simulated test in DEM (**b**) Physical test on TPA platform.

A static block factory generated and fixed multi-sphere particles with a diameter of 5 mm over a three-point bending support of 80 × 5 × 5 mm. The particles followed a normal distribution with a mean and standard deviation of 1 and 0.1 (Fig. 3a). A particle’s velocity and kinetic energy were allowed to decrease until they were less than 0.05 m/s and 0.01 J/s, respectively, before being allowed to settle over the two supports. The compressor geometry was given a downward linear translation velocity with a variable downward speed for the probe.

### Physical three-point bending test

The test was organized to govern the maximum force to bend the straw up to a certain deformation. The straw length was 80 mm and rested on steel supports (Fig. 3b). A texture analyzer (TA-XT Plus, Stable Micro Systems Ltd., Surrey, UK) with a 490 N load cell capacity was used to conduct the TPA. The test was conducted using the A/3PB probe. Three different levels of moisture content and load rates were used to examine the samples. Three loading rates were used in a bending test to assess the force at the failure region of the straw.

### DEM calibration for straw using CCD

After identifying the most dominant factors through DSD in JMP software, which can handle multiple factors simultaneously, the optimization (calibration) phase was carried out using another DOE method, Central Composite Design (CCD) by Design Expert 13 Software. Actual bending force at three moisture contents was obtained during the physical test and kept as the targeted value for optimizing straw DEM properties.

### Model validation

**Figure 4:**
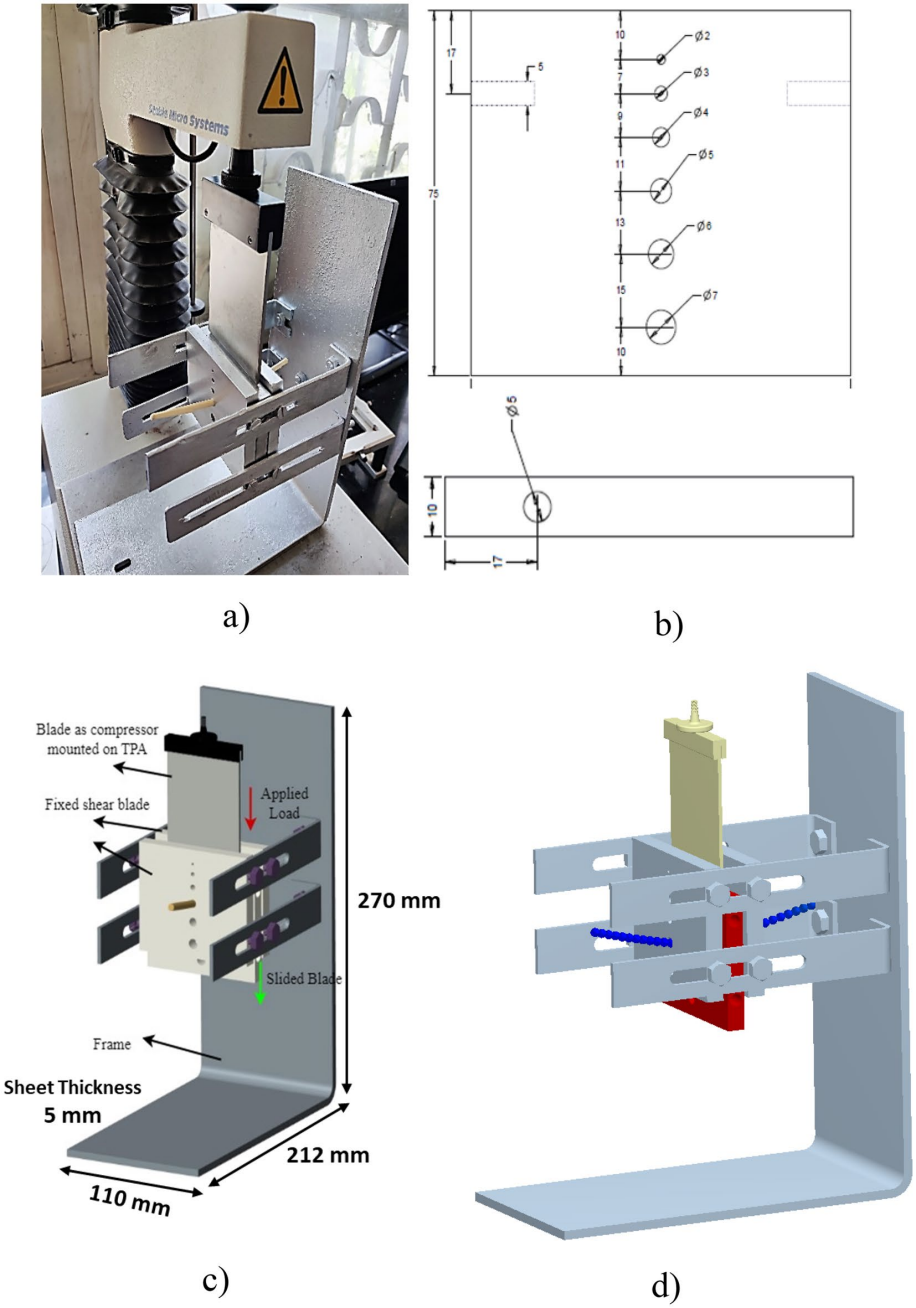
Shear strength measurement (**a**) Physical shear plate setup (**b**) dimensions of shear plates, mm and (**c**) labelled CAD model of shear plate setup (**d**) simulated shear plate setup.

A Shear Plate Test (SPT) validated the established DEM model. The dimensions of the shear plate used for the experiment are represented in Fig. 4. Comparisons were made between the shear force detected during the physical test and the DEM estimates for the shear force detected through the simulation test.

#### Shear test apparatus

A force–time deformation graph was developed during the experiment, and textural parameters such as shear strength (SS) (Eq. 6) were found with the support of software and the apparatus mentioned in the above section. The shear plate setup consisted of three rectangular plates (The outer plate is fixed, and the center plate is movable) of aluminium material (90 × 75 × 10 mm) having holes of 2–7 mm diameter. The aluminium plates were spaced 5 mm apart^26^. The force was measured using TPA when attached to a Warner Bratzler (HDP/BSW) probe to push the sliding plate of the shear plate setup. The data was recorded in TPA, and the force–time deformation graph was plotted on the screen during the experiment. A similar shear plate test was used to determine the shear strength properties of straw^27^.

### Shear plate test DEM simulation

The shear plate test’s CAD model (Fig. 4c) was converted into software with exact replicas of the real experiment’s dimensions. The shear plate experiment was then modelled using the EDEM 2021 program on a computer with an HP Intel Core i9-9900K CPU running at 3.6 GHz and 32 GB of RAM. A similar length of DEM straw model was generated in the block factory inside the hole of the shear plate by adding the spheres as Meta particles to 80 mm length of straw (Fig. 4d). The calibrated model parameters provided in the table were used to run the simulations. The particles were allowed to settle before the simulations started until their velocities and kinetic energies, respectively, fell below 0.05 m s^−1^ and 0.01 J. Using a four-core CPU solver, each simulation in the validation study was run once with a timestep of 2.19 × 10^−5^.

The operating conditions (i.e., downward velocity) are similar to the real experiment and kept in the simulated experiment. Outer two plates were fixed while the middle plate was given the linear translation motion towards the downside at certain deformation as experienced in the physical experiment. The correlation between the simulated and actual bending force was evaluated using relative error. The following formula was used to compute the relative error.

The following formula from Eq. (8) was used to compute the relative error.

8 $${\text{RE}}_{{{\text{Shear}} {\text{force}}}} = \left| {\frac{{{\text{SF}}_{{\text{o}}} - {\text{SF}}_{{\text{s}}} }}{{{\text{SF}}_{{\text{O}}} }}} \right| \times 100$$

where $RE_{Shear force}$ is the relative error (%), $SF_{O}$ and $SF_{s}$ are the observed and simulated shear force (N).

## Results

The effects of various input parameters on the response of the bending test within the DEM model were initially investigated parametrically using the Discrete Element Method (DEM). The parameters obtained from the DEM research were then refined using an additional Design of Experiments (DOE) called the Central Composite Design (CCD), and this calibration procedure was validated using the shear plate test.

### Parameterization of DEM parameters using DSD

**Figure 5:**
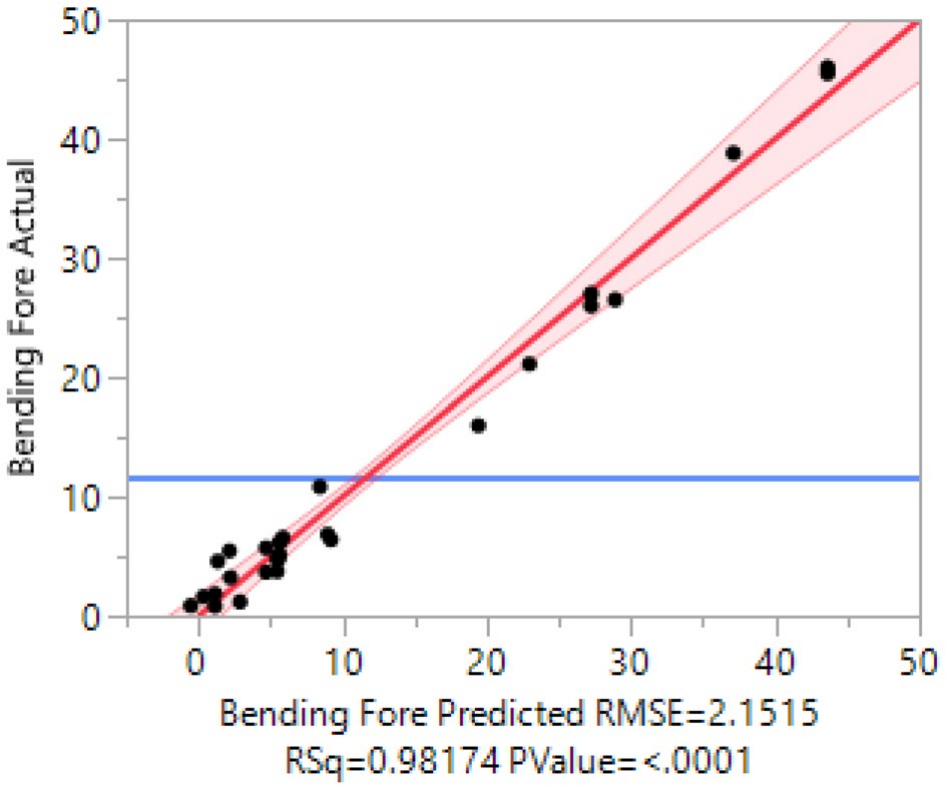
Actual versus predicted plot for bending force using DSD.

Using JMP 15 Pro, the data from the DEM simulation analysis was carried out to accomplish this goal; this strategy decomposes the response variable (Y) into two halves, indicated as Y_ME_ and Y_2nd_ and shown in Eq. (9)^28^.

9 $$Y= {Y}_{ME}+ {Y}_{2nd}$$

where Y_ME_ is the expected value of Y determined by regressing Y on the main effects and fictitious variables, Y_2nd_ is produced when Y_2nd_ = Y − Y_ME._ The determination coefficient for the model, which compares predicted values to actual values of bending force, is 0.98, whereas the RMSE is 2.151 (Fig. 5).

The results presented in Table 3 demonstrate that the main effects, including CSF (Straw-Straw), CSF (straw steel), CRF (straw steel), normal stiffness, shear stiffness, and BDS, have a highly significant impact on the bending force (*p* < 0.01). Among these parameters, BDS has the greatest influence on the bending force, with a corresponding F value of 8.42. It was followed by normal stiffness (8.28), shear stiffness (1.99), CSF straw-steel (1.67), CRF straw-steel (0.94), and CSF straw-straw (0.74). The bending force is positively impacted by all relevant factors, meaning that when their values rise, the bending force (BF) also tends to rise.

**Table 3:** Estimates of model coefficients with corresponding standard errors (SE) and *p* values for bending force (BF) using DSD.

Term	Estimate	Std error	Prob >|t|
Intercept	8.41	1.27	0.0001**
CSF straw steel	1.67	0.42	0.0008**
Normal stiffness	8.28	0.42	0.0001**
Shear stiffness	1.99	0.42	0.0001**
BDS	8.42	0.42	0.0001**
Normal stiffness * Shear stiffness	2.39	0.45	0.0001**
Normal stiffness * BDS	6.65	0.44	0.0001**
Shear stiffness * BDS	2.13	0.44	0.0001**
BDS * BDS	3.65	1.35	0.0139*

**Significant at 1% level of significance, *significant at 5% level of significance, *NS* non-significant.

**Figure 6:**
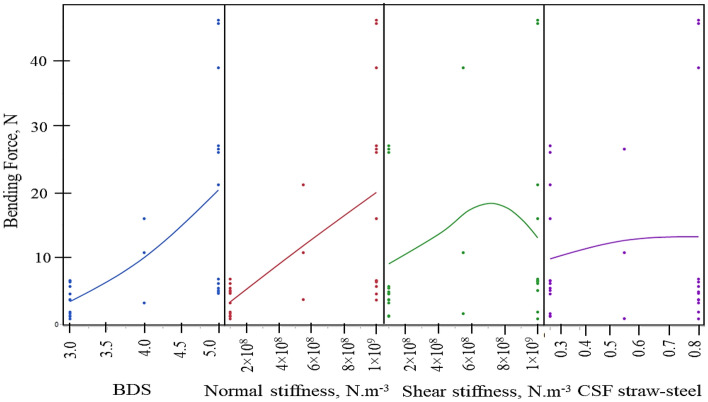
Effect of various DEM contact parameters on Bending force (BF).

The BDS of straw has a significant effect on the bending force (BF). Increasing the BDS value from 3 to 5 results in an increase in the bending force. Similarly, the Normal stiffness has a linear relationship with the bending force, where an increase in the Normal stiffness from 1 × 10^8^ to 1 × 10^9^ leads to an increase in the bending force (Fig. 6).

Regarding shear stiffness, initially increasing the shear stiffness from 1 × 10^8^ to 7 × 10^8^ increases the bending force. However, further increasing the shear stiffness from 7 × 10^8^ to 1 × 10^9^ decreases the bending force. The CSF of straw steel also significantly affects the bending force. Initially, as the CSF value increases from 0.3 to 0.55, the bending force also increases. However, beyond the value of 0.55, the bending force tends to remain relatively constant.

### Calibration of DEM parameters of paddy straw model using CCD

Based on the findings outlined in Table 3, it can be seen that only four DEM factors have a significant influence on the BF. These factors are CSF (straw-steel), Normal stiffness, Shear stiffness, and BDS. The shear modulus of the straw was kept constant at 1 × 10^6^ Pa because, beyond this limit, the straw may become unstable during the simulation^29^.

Table 4 gives information on four factors: their equivalent levels for CSF (straw-steel), Normal stiffness, Shear stiffness, and Bonded disc scale. There are three levels at which each of these parameters is defined. The Central Composite Design (CCD) matrix and the information gathered from the related trials are shown in Table 5. The model fit for the bending test using CCD and the data points line up nicely along a straight line, supporting the model’s normality assumption.

**Table 4:** DEM input parameters for the straw calibration using the CCD experiment.

Parameters	Units	Symbols	Levels		
			Low (− 1)	Middle (0)	High (+ 1)
Coefficient of static friction (straw-steel)	–	CSF straw steel	0.3	0.55	0.8
Normal stiffness	N m^−3^	NS	1 × 10^8^	5.5 × 10^8^	1 × 10^9^
Shear stiffness	N m^−3^	SS	1 × 10^8^	5.5 × 10^8^	1 × 10^9^
Bonded disk scale	–	BDS	3	4	5

**Table 5:** Experimental design matrix for CCD.

Run	CSF straw-steel	Normal stiffness	Shear stiffness	BDS	Bending force
1	0.55	1E+07	5.05E+08	4	0.14
2	0.8	1E+07	1E+07	3	0.0391
3	0.55	5.05E+08	1E+09	4	4.57
4	0.3	1E+07	1E+09	5	0.291
5	0.3	1E+09	1E+09	5	6.8
6	0.8	1E+07	1E+07	5	0.264
7	0.8	1E+09	1E+09	3	2.94
8	0.8	5.05E+08	5.05E+08	4	4.5
9	0.55	5.05E+08	5.05E+08	3	1.534
10	0.8	1E+09	1E+09	5	5.78
11	0.3	1E+07	1E+09	3	0.043
12	0.3	1E+07	1E+07	5	0.256
13	0.8	1E+09	1E+07	5	4.48
14	0.3	1E+09	1E+09	3	2.84
15	0.55	5.05E+08	5.05E+08	5	5.26
16	0.55	5.05E+08	5.05E+08	4	4.48
17	0.3	1E+09	1E+07	5	4.72
18	0.3	1E+09	1E+07	3	1.16
19	0.3	1E+07	1E+07	3	0.038
20	0.8	1E+09	1E+07	3	1.222
21	0.55	5.05E+08	1E+07	4	1.929
22	0.8	1E+07	1E+09	3	0.0447
23	0.3	5.05E+08	5.05E+08	4	4.38
24	0.55	5.05E+08	5.05E+08	4	4.48
25	0.8	1E+07	1E+09	5	0.308
26	0.55	1E+09	5.05E+08	4	4.27

The selected model’s adjusted and predicted determination coefficient values were 0.99 and 0.98, respectively, and their difference was less than 0.20. This confirms the adequacy of the selected model to describe the effect of independent variables. The showing ANOVA for bending force using CCD. The R^2^ value of 0.99 indicates good agreement between independent parameters and bending force. Table 6 indicates that normal stiffness, shear stiffness, and BDS significantly affected bending force at 1% significance level. The created model was statistically significant (*p* < 0.001), as shown in Table 6, indicating that lack of fit is insignificant. As a result, it can be concluded that the constructed model has excellent statistical quality. Except for CSF (straw-steel) with Normal stiffness, Shear stiffness, and BDS, all of the main and interaction effects favourably impacted the response.

**Table 6:** ANOVA for the bending force using CCD.

Source	Sum of squares	df	Mean square	F-value	*p*-value
Model	81.29	14	5.81	221.34	0.0001**
A-CSF	0.01	1	0.01	0.01	NS
B-N stiffness	51.63	1	51.63	1967.98	0.0001**
C-S stiffness	0.80	1	0.80	30.51	0.0002**
D-BDS	9.60	1	9.60	365.85	0.0001**
AB	0.01	1	0.01	0.19	NS
AC	0.01	1	0.01	0.02	NS
AD	0.01	1	0.01	0.18	NS
BC	0.21	1	0.21	8.18	0.0155*
BD	0.72	1	0.72	27.67	0.0003**
CD	0.08	1	0.07	3.03	NS
R^2^	0.99				
Residual	0.2886	11	0.0262		
Lack of fit	0.2886	10	0.06 (NS)		
Pure error	0.0000	1	0.0000		

**Significant at 1% level of significance, *significant at 5% level of significance, *NS* non-significant.

The interaction terms BC and BD had a significant effect on the bending force at 5% and 1% level of significance, respectively. In order to obtain the reduced polynomial model, insignificant terms were ignored from the quadratic model was represented in the Eq. (10).

10 $$\begin{aligned} {\text{ln}}\left( {{\text{BF}}} \right){ = } & - 0.{21} + {1}.{14} \times {1}0^{{ - {8}}} \times {\text{B}} + {1}.{73} \times {1}0^{{ - {9}}} \times {\text{BC}} + {3}.{26} \times {\text{D}} + {4}.{72} \times {1}0^{{ - {19}}} \times {\text{BC}}{-}{4}.{3}0 \times {\text{BD}}{-}{1}.{42} \times {1}0^{{ - {1}0}} \\ & \times \;{\text{CD}}{-}{6}.{43} \times {1}0^{{ - {1}0}} \times {\text{B}}^{{2}} {-}{9}.{43} \times {1}0^{{ - {19}}} \times {\text{C}}^{{2}} {-}0.{27} \times {\text{D}}^{{2}} \\ \end{aligned}$$

The physical three-point bending test results showed that the maximum bending force was 2.6 N, 3 N, and 5.5 N at a loading rate of 0.42 mm s^−1^ applied on straw specimens representing moisture levels of M_1_, M_2,_ and M_3_, respectively (Table 7). The moisture content was mimicked in the DEM simulation by adjusting the bonding parameters while keeping the other parameters constant.

**Table 7:** Calibrated parameters of the straw specimen using CCD.

Moisture content, %	CSF straw—steel	Normal Stiffness, N m^−3^	Shear Stiffness, N m^−3^	BDS	Target force
M_1_ (35 ± 3.4%)	0.363	7.51 × 10^8^	1.02 × 10^8^	3.33	2.63
M_2_ (24 ± 2.2%)	0.363	3.36 × 10^8^	2.94 × 10^8^	4.92	3
M_3_ (17 ± 2.6%)	0.363	4.44 × 10^9^	8.30 × 10^8^	4.99	5.57

**Figure 7:**
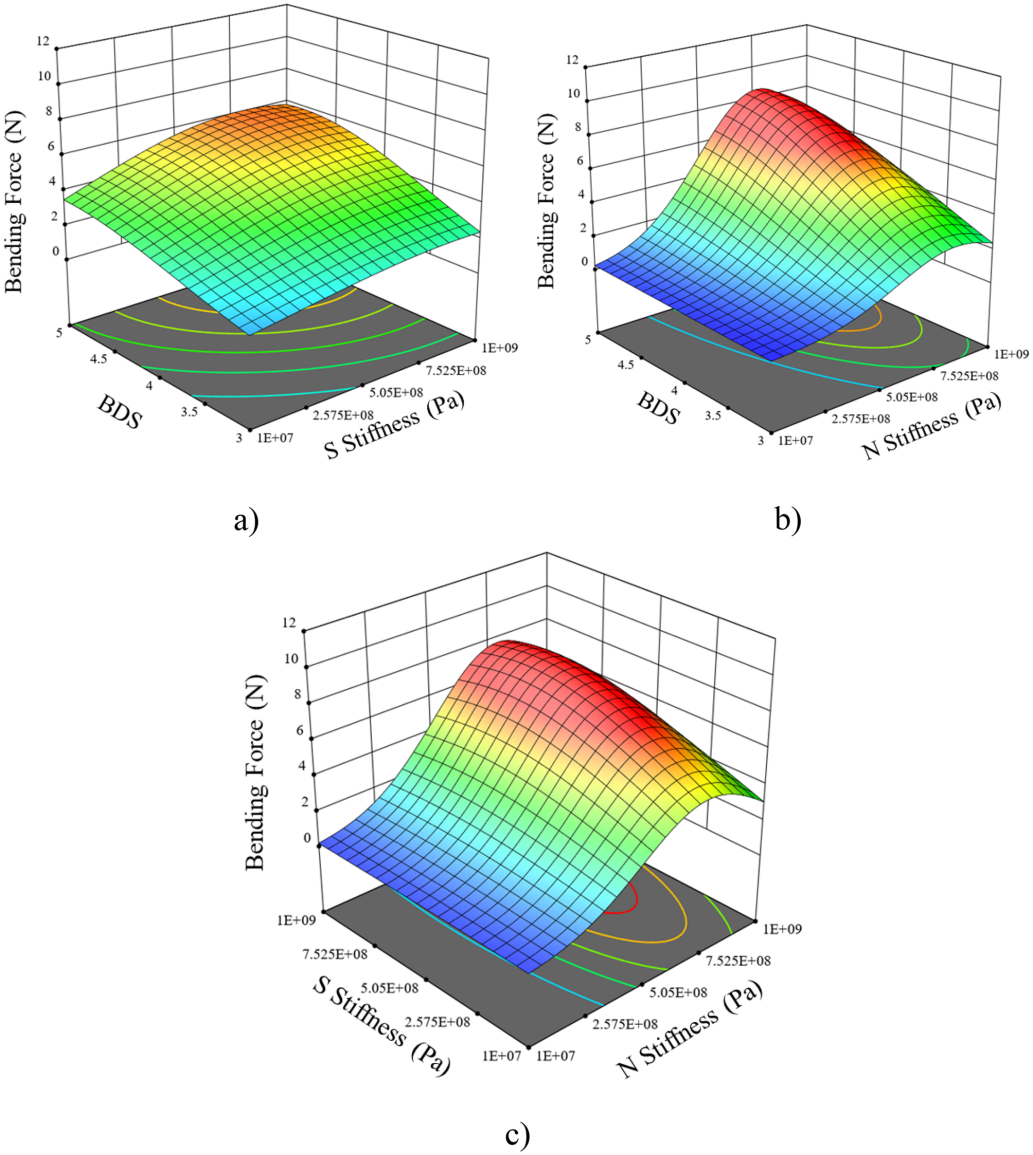
Response surface of bending force (BF) (**a**) BDS and shear stiffness (**b**) shear stiffness and normal stiffness (**c**) bonded disk scale (BDS) and normal stiffness.

The maximum and minimum values of the bending force (BF) were found to be 6.8 and 1.15 N, respectively. The BF exhibited an increasing trend with an increase in shear stiffness up to 7.52 × 10^8^ N m^−3^, after which it decreased within the range of variables. A similar trend was observed for BDS. The BF linearly increased from 2.8 to 6.8 N with an increase in BDS (Fig. 7a) in the range of 3–5 at a shear stiffness of 1 × 10^9^ N m^−3^.

Similarly, it increased from 4.4 N to 6.8 N at BDS 5 with an increase in shear stiffness ranging from 1 × 10^7^ to 1 × 10^9^ N m^−3^ (Fig. 7b). The BF increased with an increase in normal stiffness up to 6.8 × 10^8^ N m^−3^ after which it decreased. The BF exhibited a relatively constant trend with an increase in BDS ranging from 3 to 5. The BF followed a concave-shaped curve, first increasing and then slightly decreasing within the normal stiffness range of 1 × 10^7^ to 1 × 10^9^ N m^−3^ at the shear stiffness range of 1 × 10^7^ to 1 × 10^9^ N m^−3^ (Fig. 7c). A similar trend was observed for shear stiffness while keeping the maximum value of Normal stiffness. The calibrated parameters of straw stem at 35 ± 3.4% moisture content and other selected values of the straw model were presented in Table 8.

**Table 8:** Calibrated set of DEM parameters for developed straw stem (35 ± 3.4% moisture content).

Sr. No	DEM parameter	Unit	Value	References
1	Solid density	kg.m^-3^	1325	^16^
2	Shear modulus (straw)	Pa	1 × 10^6^	Selected
3	Shear modulus (steel)	Pa	7.9 × 10^10^	^16^
4	Coefficient of restitution (straw-straw)	–	0.2	^30^
5	Coefficient of sliding friction (straw-straw)	–	0.3	^31^
6	Coefficient of rolling friction (straw-straw)	–	0.4	^30^
7	Coefficient of restitution (straw-steel)	–	0.2	^32^
8	Coefficient of sliding friction (straw-steel)	–	0.35	^33^
9	Coefficient of rolling friction (straw-steel)	–	0.01	^32^
10	Normal stiffness	N m^−3^	7.5 × 10^8^	Calibrated
11	Shear stiffness	N m^−3^	1.02 × 10^8^	Calibrated
12	Bonded disc scale	–	3.33	Calibrated
13	Time step	s	10% of Rayleigh time step	

### Model validation

**Figure 8:**
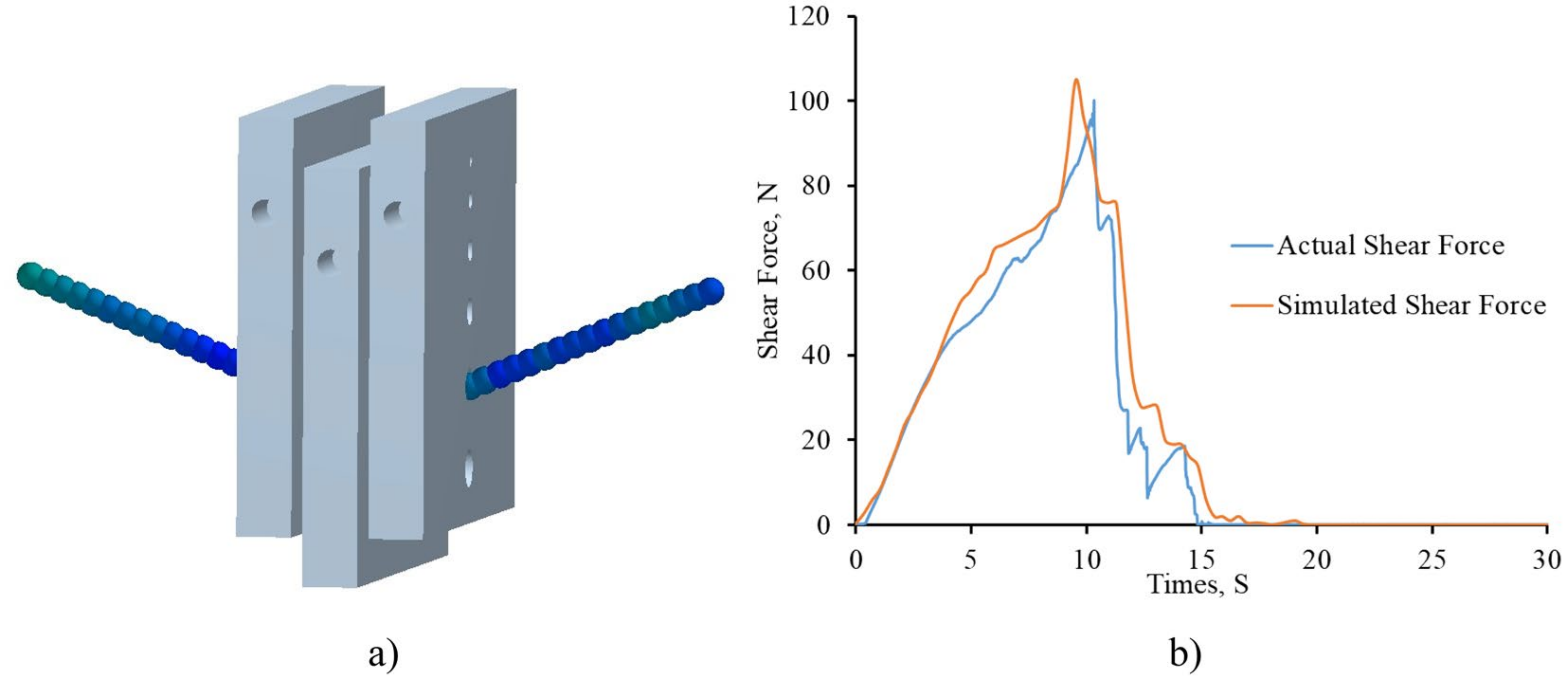
(**a**) Simulation of shear plate test in EDEM software (**b**) comparison of simulated and actual shear force.

**Figure 9:**
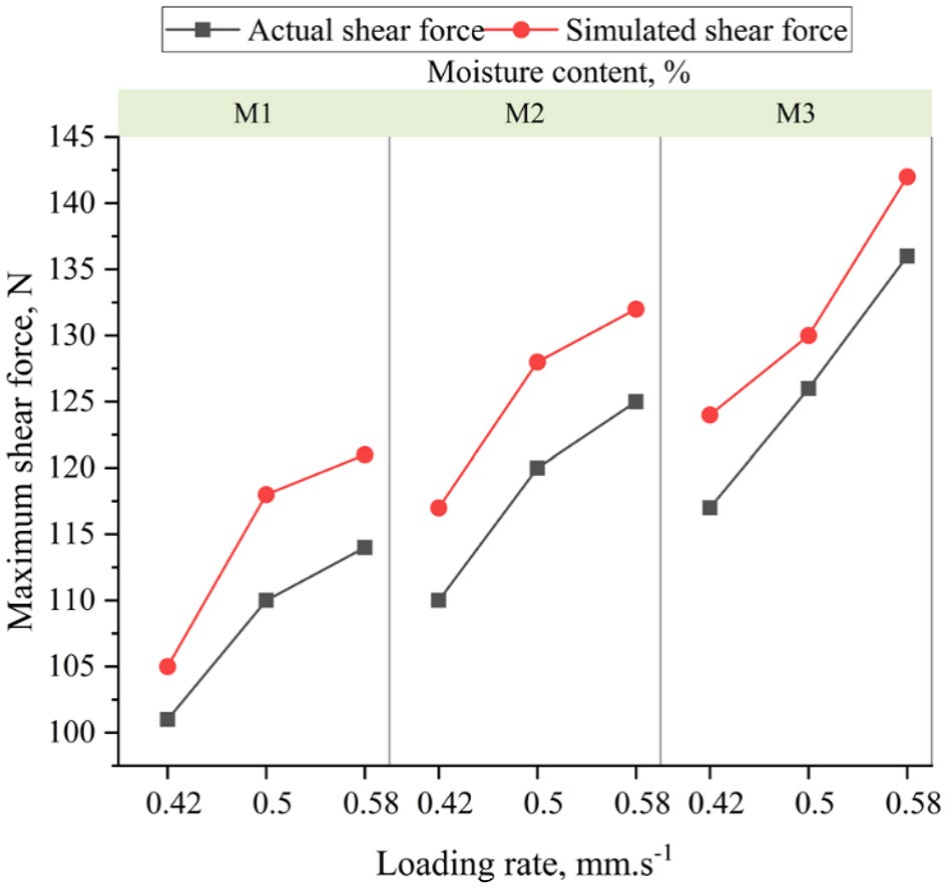
Relationship between an actual and simulated shear force of paddy straw stem.

The method outlined in Section "DEM calibration for straw using CCD" was used to validate the constructed DEM straw model (Table 8). The simulated shear force at different loads, such as 0.42, 0.5, and 0.58 mm s^−1^ with the same deflection depth, is depicted in Fig. 8, and it shows good agreement with the physical shear test. A well-calibrated model shows a similar trend (Fig. 8) for a maximum shear force at the abovementioned conditions with minimum relative error (Table 9). The maximum physical shear force was measured to be 101.8 N, while the maximum simulated shear force was 104.4 N at moisture content M_1_ and applied a loading rate of 0.42 mm s^−1^. Similar values were observed for other combinations of moisture contents and loads, as depicted in Fig. 9. The highest simulated shear force value of 143 N was obtained at moisture content M3 at a loading rate of 0.58 mm s^−1^.

**Table 9:** Observed and simulated shear force at different loading rates and moisture content with corresponding relative error.

Loading rate (mm s^−1^)	Moisture content (%)	Observed shear force (N)	Simulated shear force (N)	Relative error (%)	Average relative error (%)
0.42	M_1_	101	105	3.96	5.43
0.42	M_1_	110	117	6.36	
0.42	M_1_	117	124	5.98	
0.5	M_2_	110	118	9.09	7.63
0.5	M_2_	120	128	6.66	
0.5	M_2_	126	130	7.14	
0.58	M_3_	114	121	9.56	8.86
0.58	M_3_	125	132	10.40	
0.58	M_3_	136	142	6.61

**Figure 10:**
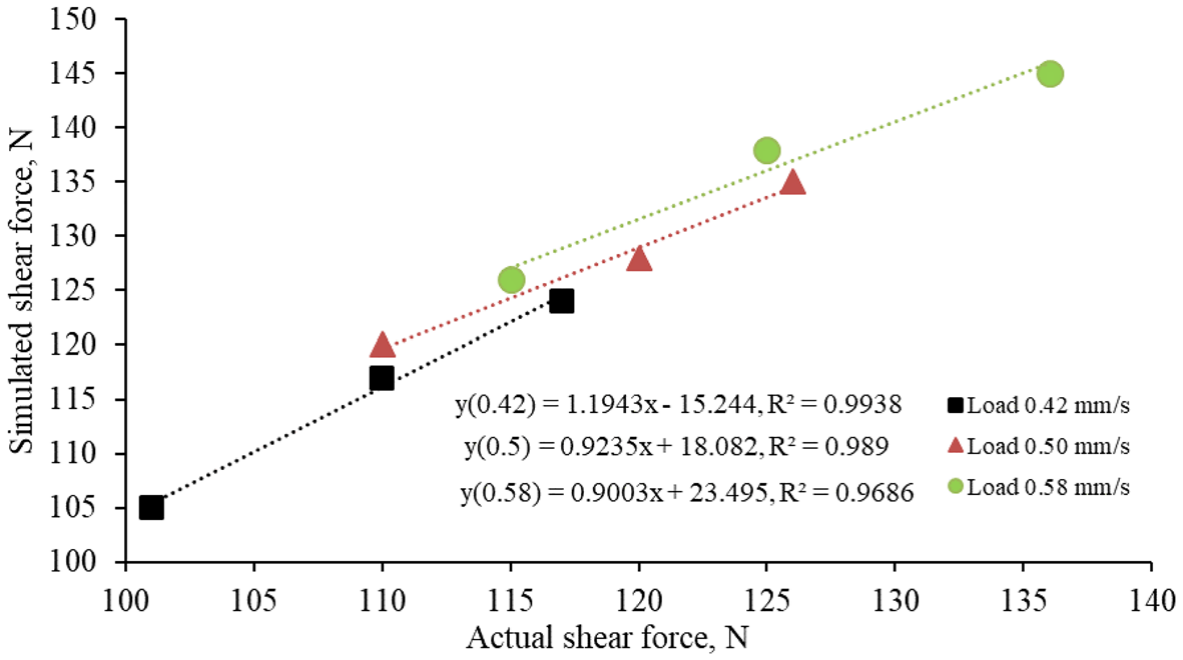
Actual versus simulated values of shear force.

The loading rates of 0.42 mm s^−1^, 0.50 mm s^−1^, and 0.58 mm s^−1^ resulted in maximum shear forces with average relative errors of 5.43, 7.63, and 8.86%, respectively (Table 9). The graph (Fig. 9) shows the simulated versus actual shear force at the loading rate of 0.42, 0.5, and 0.58 mm s^−1^ with an excellent correlation R^2^ value of 0.99, 0.98, and 0.96, respectively (Fig. 10).

## Discussion

Sensitivity analysis for the DEM straw model identified key parameters, given the challenge of handling numerous parameters in a single experiment^25^. Therefore, parameters with non-significant influence should be screened out first before proceeding with the actual experiment^34^. Parameterization of the DEM model revealed that bonded disc scale (BDS), normal stiffness and shear stiffness were the most influential parameters. BDS and Normal stiffness affect the bending force, whereas the bending force initially increases with increasing the shear stiffness (1 × 10^8^ to 7 × 10^8^ N m^−3^) and then decreases with further increasing the value of shear stiffness (7 × 10^8^ to 1 × 10^9^ N m^−3^). BDS affects linearly on the bending force; when the bonded disc scale is increased, the contact area between the straw particles and the other particles in the DEM is increased, resulting in an increase in bending force^7,9^. Higher normal stiffness means that it will be more difficult to compress the material, leading to a higher bending force. This is because when the disk contacts the straw, the straw resists compression, which causes an increase in the bending force. Higher shear stiffness means that it will be more difficult to deform the material, leading to a higher bending force^16^. The optimized (calibrated) values of DEM parameters at three different moisture content. The straw model was validated by the shear plate test setup with three different loading rates. Maximum and minimum bending force and shear force were varied from 2.3 to 8.9 N and 101.8 to 143 N^35^. The relative error between the actual and simulated model is less than 10 per cent under the acceptable limit and represents the real behaviour of straw^12^.

## Conclusions

Two DOE techniques—namely, a Definitive Screening Design (DSD) for screening and a Central Composite Design (CCD) for calibration—were employed to parameterize the model parameters of the Hertz-Mendlin and parallel contact models to calibrate a flexible paddy straw. The following parameters: Normal stiffness, shear stiffness, and bonded disc scale were seen in CCD, which greatly affected the bending force during the simulated bending test with a coefficient of determination near unity (R^2^ = 0.9965). The flexible straw could withstand normal and tangential displacements, which enabled the simulation of crop straw’s deformation characteristics. When using the best bonding parameters in conjunction with moisture content, M_1_, M_2,_ and M_3_ were calibrated and validated to be (normal stiffness per unit area: 5.05 × 10^8^, 5.05 × 10^8^ and 1 × 10^9^ N m^−3^, Shear stiffness per unit area: 5.05 × 10^8^, 9.33 × 10^8^ and 5.05 × 10^8^ N m^−3^, BDS 3.34, 3.6 and 3.65). The relative error of the average of maximum actual and simulated bending force at different load rates and moisture content was 10.4, 4.25, and 7.2%; similarly, for shear force, it varies from 5.43, 7.63, and 8.86%, respectively. It depicts the accuracy of the regression model used to predict the bending and shear force. The flexible paddy straw described in this study can be implemented in DEM models to simulate the interactions between straw particles and equipment. Similarly, other crops can benefit by adopting modelling and parameter calibration techniques.

## Data Availability

The corresponding author can provide the datasets created and analyzed during the current investigation upon reasonable request.
